# Special Diseases from Mental Strain

**Published:** 1870-09

**Authors:** 


### Special Diseases from Mental Strain.

According to B. W. Richardson, M. D. (*Am. Jour. of Insanity*) dseases following upon mental shock or strain, are divisible into two classes. There is a *primary* class in which the mental shock stands out as the direct and only cause of the malady, and there is a *secondary* class in which the mental shock or strain appears only to increase or exaggerate symptoms of disease which pre-existed.

In the first class the diseases produced are the same as those which sometimes follow upon the receipt of physical injury to the nervous centres. The most distinct forms of disease of this nature with which he is familiar are, diabetes, paralysis (local or general), intermittent pulse, and arterial relaxation with arterial murmur. Diabetes from sudden mental shock is a true type, a pure type, of a physical malady of mental origin.

The class of cases where the symptoms due to nervous mischief are secondary, include, according to his views, syphilis, some chronic eruptions on the skin (psoriasis especially), cancer, epilipsy, and insanity itself.

Thus the symptoms of tertiary syphilis will recur on venereal excess, without any introduction of new venereal poison; thus eruption on the skin will recur from nervous shock; thus cancer so frequently shows the first signs of its presence in mental anxiety; and in two cases of persons predisposed to epilepsy, the first seizure was clearly traced to mental prostration.—*Med. Record.*

